# Targeted Next-Generation Sequencing of Circulating Tumor DNA Mutations among Metastatic Breast Cancer Patients

**DOI:** 10.3390/curroncol28040214

**Published:** 2021-06-24

**Authors:** Min-Ying Sun, Fang-Qin Lin, Lu-Jia Chen, Hong Li, Wei-Quan Lin, Hong-Yan Du, Xue-Xi Yang, Ming Li

**Affiliations:** 1 Institute of Antibody Engineering, School of Laboratory Medicine and Biotechnology, Southern Medical University, Guangzhou 510515, China; sunmy1220@163.com (M.-Y.S.); gzduhongyan@126.com (H.-Y.D.); 2 GZMU-GIBH School of Life Sciences, Guangzhou Medical University, Guangzhou 511436, China; linfangqin@gzhmu.edu.cn; 3 Nanfang Hospital, Southern Medical University, Guangzhou 510515, China; dr_chenlj@smu.edu.cn (L.-J.C.); lhnfyy@163.com (H.L.); 4 Department of Primary Public Health, Guangzhou Center for Disease Control and Prevention, Guangzhou 510440, China; linweiquan0503@163.com; 5 Institute of Public Health, Guangzhou Medical University & Guangzhou Center for Disease Control and Prevention, Guangzhou 510440, China

**Keywords:** circulating tumor DNA, mutation, breast cancer, targeted next-generation sequencing

## Abstract

Liquid biopsy through the detection of circulating tumor DNA (ctDNA) has potential advantages in cancer monitoring and prediction. However, most previous studies in this area were performed with a few hotspot genes, single time point detection, or insufficient sequencing depth. In this study, we performed targeted next-generation sequencing (NGS) with a customized panel in metastatic breast cancer (MBC) patients. Fifty-four plasma samples were taken before chemotherapy and after the third course of treatment for detection and analysis. Paired lymphocytes were also included to eliminate clonal hematopoiesis (CH)-related alternatives. A total of 1182 nonsynonymous mutations in 419 genes were identified. More ctDNA mutations were detected in patients with tumors > 3 cm (*p* = 0.035) and HER2(−) patients (*p* = 0.029). For a single gene, the distribution of ctDNA mutations was also correlated with clinical characteristics. Multivariate regression analysis revealed that HER2 status was significantly associated with mutation burden (*OR* 0.02, 95% *CI* 0–0.62, *p* = 0.025). The profiles of ctDNA mutations exhibited marked discrepancies between two time points, and baseline ctDNA was more sensitive and specific than that after chemotherapy. Finally, elevated ctDNA mutation level was positively correlated with poor survival (*p* < 0.001). Mutations in ctDNA could serve as a potential biomarker for the evaluation, prediction, and clinical management guidance of MBC patients with chemotherapy.

## 1. Introduction

Breast cancer is the most common cancer among women in China as well as worldwide [1]. With progress in cancer management, most patients diagnosed in early stages can be treated satisfactorily by surgical resection and adjuvant therapies [2]. However, metastatic breast cancer (MBC) is still a challenge for clinicians due to the development of drug resistance, and a decreased overall survival rate is closely linked to the incidence of distant metastases [3,4]. MBC is a heterogeneous and dynamic disease with a range of biological characteristics, genomic alterations, and clinical outcomes [5]. It is important to monitor the clinical progression and therapy responsiveness during personalized treatment [6]. Tumor DNA testing and analysis can provide a current assessment of mutational profile to guide clinical therapy, although these are limited by geographical and temporal heterogeneity [7]. Recent studies demonstrated that circulating tumor DNA (ctDNA) released from tumor cells into blood circulation, which contains a great deal of genetic and epigenetic information associated with cancer, is a promising biomarker to estimate cancer prognosis and the efficacy of treatment [8,9,10,11].

Monitoring of ctDNA can be performed more easily, repeatedly, and is noninvasive compared with that of tissue biopsy [12]. Furthermore, mutation information from all metastatic sites is included in ctDNA, so it may reflect tumor heterogeneity more completely [13]. Studies confirmed that ctDNA sequencing could offer accurate, rapid genomic profile of MBC that enables the selection of mutation-directed therapies for patients [14,15]. However, there are many scientific and logistical challenges for clinical applications of ctDNA, such as the low level of ctDNA in the “sea” of cell-free DNA (cfDNA) and the small number of validated ctDNA-based driver genes or mutations specific for MBC other than *ER*, *HER2*, *PIK3CA*, and *AKT1* [16]. In addition, most studies were performed with detection of single sampling or insufficient sequencing depth due to technical and cost limitations [17]. More data of high quality and comprehensive monitoring are essential to support and promote the application of ctDNA for the evaluation of MBC.

In this prospective study, we designed a personalized target-capture region associated with breast cancer. Plasma samples obtained at two time points were examined by ultrasensitive, high-throughput next-generation sequencing (NGS). Meanwhile, parallel sequencing from paired lymphocytes was performed to filter out interference by clonal hematopoiesis (CH) variants. Analyses and comparisons were performed to identify ctDNA mutations related to MBC progression and clinical outcome. The results will provide evidence regarding the clinical utility of ctDNA and potentially explore the molecular mechanism of MBC.

## 2. Materials and Methods

### 2.1. Study Subjects and Blood Collection

Patients with pathologically diagnosed MBC between February 2017 and October 2019 were enrolled in the study. The inclusion criteria were Han Chinese women less than 70 years old who agreed to participate in this study and share their clinical information. The exclusion criteria were a previous cancer diagnosis within the last five years and an inability to provide adequate blood samples for NGS or undergo medical followup. A total of 27 patients receiving consecutive gemcitabine and capecitabine treatment [18] (trastuzumab was combined for patients with HER2-positive) were included in the study population. Followup was performed to estimate the association between ctDNA profile and prognosis. Clinical data were obtained from the medical records and followup results. Therapeutic effects and prognosis were evaluated according to Response Evaluation Criteria in Solid Tumors guidelines after three courses of treatment.

Peripheral blood samples were collected in 10-mL Streck tubes at two time points: before the initiation of chemotherapy and the end of the third course of treatment (63 ± 6 days). Samples were shipped at 4–8 °C to the laboratory and processed within 3 h. Plasma (4–5 mL) and lymphocytes (150–200 μL) were isolated by centrifugation (1600× *g*, 4 °C for 10 min, and then 16,000× *g*, 4 °C for 10 min) immediately and stored at −80 °C.

### 2.2. DNA Extraction and Assessment

The cfDNA and genomic DNA (gDNA) were extracted from plasma and lymphocytes, respectively, using a QIAamp Circulating Nucleic Acid Kit (Qiagen, Valencia, CA, USA) and QIAamp DNA Blood Mini Kit (Qiagen), according to the manufacturer’s instructions. The DNA concentration was quantified using a Qubit 2.0 Fluorometer with Qubit dsDNA High Sensitivity (HS) Assay Kit (Fisher Scientific, Newark, DE, USA). An Agilent 2100 Bioanalyzer and DNA HS Kit (Agilent Technologies, Santa Clara, CA, USA) were used to assess the fragment length distribution. Only samples containing > 20 ng of cfDNA and >500 ng of gDNA were processed for further analysis. All 81 DNA samples (54 cfDNA and 27 gDNA) were prepared and stored at −20 °C before library construction.

### 2.3. Gene Panel Design and Target-Capture Sequencing

In this study, a personalized target panel was designed according to NCBI Build 37.1/GRCh37. Genomic exons that are frequently mutated in breast cancer or related to chemotherapy, immunotherapy, and targeted drugs were identified by searching the COSMIC database, The Cancer Genome Atlas, and published studies. An iterative algorithm was applied to maximize the number of mutations per patient while minimizing the region size. Target panel selection involved tradeoffs between sequencing cost, sensitivity, and coverage. Target-capture hybrid probes were custom designed through the NimbleGen Design portal (v1.2.R1, Roche, Basel, Switzerland).

DNA libraries were prepared with a KAPA HyperPrep Kit (Kapa Biosystems, Woburn, MA, USA), including end repair and A-tailing, adapter ligation, postligation cleanup, library amplification, and postamplification cleanup. The DNA polymerase displayed strong 3′→5′ exonuclease activity and a low error rate. Agencourt AMPure XP beads (Beckman Coulter, Fullerton, CA, USA) were employed for “with-bead” enzymatic and cleanup steps. Aliquots of 20–30 ng of plasma DNA were used directly for library construction. For gDNA, 500–1000 ng DNA was sheared with a Covaris S2 instrument (Boston, MA, USA) set for 200-bp fragments and then used for library construction.

Groups of four libraries were incorporated in a single capture pool. Hybridization and target capture were performed with custom probes from the SeqCap EZ Choice Library (Roche). The captured DNA products were amplified and purified with NimbleGen SeqCap Kits (Roche). After quality control of the DNA concentration and fragment distribution, we applied targeted NGS using 2 × 75 bp or 2 × 150 bp paired-end reads on the MiSeq Sequencing system (Illumina, San Diego, CA, USA) including a negative control in accordance with the manufacturer’s recommendations.

### 2.4. Data Processing and Statistical Analysis

Software fastQC (version 0.11.9, Babraham Institute, Cambridge, UK) was used for sequencing data quality control. Low-quality data, including N bases ≥ 50%, proportion of bases with a Phred quality score ≤ 15 above 80%, or read length ≤ 30 bp, were excluded. The terminal adaptor sequences were removed with cutadapt (version 3.0, National Bioinformatics Infrastructure Sweden, Uppsala, Sweden). The reads were then mapped to the hg19 reference human genome using Burrows–Wheeler Aligner (BWA, version 0.7.17, Wellcome Trust Sanger Institute, Cambridge, UK). Genome Analysis Toolkit (GATK, version 4.1.6, Broad Institute, Cambridge, MA, USA), Picard (version 2.22.3, Broad Institute), and SAMtools (version 1.10, Wellcome Trust Sanger Institute) were used to call small insertions/deletions (indels) and mutations according to the COSMIC and dbSNP databases. Annovar (2020Apr01, University of Pennsylvania, Philadelphia, PA, USA) was used for annotation with multiple databases.

Variations were filtered out if they had low depth < 2000× in cfDNA or <1000× in gDNA, they were supported by less than five high-quality sequencing reads for cfDNA and two high-quality reads for gDNA, or if they were synonymous variants, including single-nucleotide variants (SNVs) and indels. Variants detected in the plasma DNA that were wild type in the gDNA were considered ctDNA mutations and included for statistical analysis.

Gene Ontology (GO) and Kyoto Encyclopedia of Genes and Genomes (KEGG) enrichment analyses of the mutated genes were performed using topGO (version 2.40.0, Max Planck Institute for Informatics, Saarbrücken, Germany), clusterProfiler (version 3.16.1, Jinan University, Guangzhou, China) and ggplot2 (version 3.3.3, Auckland University, Auckland, New Zealand). The associations between ctDNA mutations and clinical characteristics were analyzed using SPSS 22.0 (SPSS Inc., Chicago, IL, USA) and R language (version 4.0.3, R Foundation for Statistical Computing, Vienna, Austria). The endpoint of clinical observation for the survival analysis was the last followup (31 October 2019) or death. A progression-free survival (PFS) analysis was estimated by the Kaplan–Meier method using survival (version 3.2-7, Mayo Clinic, Rochester, MN, USA) and survminer (version 0.4.9, University of Montpellier, Montpellier, France). Survival curves with a hazard ratio (HR) and 95% confidence interval (CI) were plotted. The tests were two-sided, and *p* < 0.05 was taken to indicate statistical significance.

## 3. Results

### 3.1. Clinical Characteristics of the Patients and Target-Capture Sequencing

The main clinical characteristics of the 27 MBC patients included in the study are shown in Table 1. The mean age at diagnosis was 51.30 years (range, 33–68 years). Most patients had infiltrating ductal carcinoma with lymph node, bone, or hepatic metastasis. In total, 14 (51.8%) patients had a maximal tumor diameter > 3 cm, and 11 (40.74%) patients gave birth three or more times. The proportions of ER(+), PR(+), and HER2(+) cases were 62.96%, 59.26%, and 62.96%, respectively.

After running an iterative algorithm with multiple databases and optimization by the NimbleGen Design portal (Roche), we selected a custom panel covering 119.20 kb of the genome. The panel included 961 exons of 835 common driver genes distributed over all chromosomes. Details of the target-capture panel are presented in Table S1. DNA was successfully extracted from all 81 samples and qualified for target-capture sequencing. We obtained an average of 810.45 Mb (range 303.08–1424.64 Mb) and 403.28 Mb (range 183.51–789.27 Mb) of high-quality data for the cfDNA and gDNA samples, respectively. The average sequencing depths for cfDNA and gDNA were 6799× (range 2543–11,952×) and 3383× (range 1540–6621×), respectively.

### 3.2. Identification of ctDNA Mutations and Related Genes

**Figure 1:**
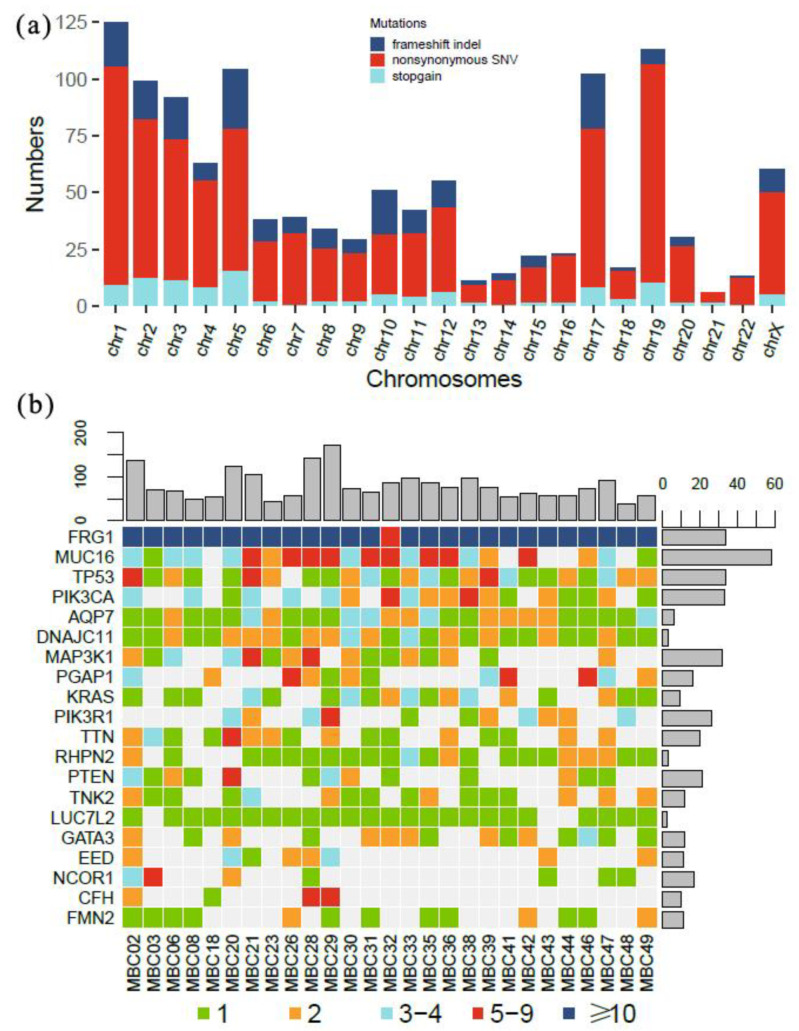
Profiles of ctDNA mutations identified. (**a**) Distribution of ctDNA mutations on all chromosomes. Height of each column represents number of mutations. (**b**) Mutation burden of ctDNA in top 20 genes for 27 MBC patients. Number of mutations in each gene and patients are listed right and top, respectively.

Some mutations originating from CH-related variants in lymphocytes can also be traced in cfDNA, which may interfere with the analysis of ctDNA. After the comparison and elimination of CH variants, we identified 1182 nonsynonymous mutations from all samples, including frameshift indels, stopgains, and SNVs. They were distributed in 419 genes on all chromosomes (as illustrated in Figure 1a). All patients had mutations in *FRG1*, *AQP7*, and *DNAJC11*. Mutations were detected most frequently in *FRG1*, and the highest mutation burden was seen in *MUC16* with 58 mutations. The top 20 genes most frequently mutated in the ctDNA are shown in Figure 1b. Some typical cancer-related genes such as *TP53*, *PIK3CA*, *MAPK3K1*, *KRAS*, and *PTEN* were also included. The number of ctDNA mutations varied markedly between patients (range 38–171, mean 79.96).

To evaluate the influence of biological features by ctDNA mutations, GO and KEGG enrichment analyses were conducted for the mutated genes from three subsets (overall, baseline, and after chemotherapy). The results of GO analysis revealed that 124, 107, and 91 genetic functions were affected in three subsets, respectively (as illustrated in Table S3). For baseline mutations, 42, 33, and 32 subjects were significantly related to biological process (BP), cellular component (CC), and molecular function (MF), respectively. The numbers were 33, 38, and 20 for mutations after chemotherapy. The top ten subjects of each part were selected for further comparison and analysis according to statistical significance. It revealed that 2, 7, and 6 subjects were coaltered in all three subsets and correlated with BP, CC, and MF, respectively, including regulation of cellular component size, cation channel complex, and calmodulin binding. There were more discrepancies among the three subsets according to BP enrichment than that of CC and MF (as illustrated in Figure S1).

**Figure 2:**
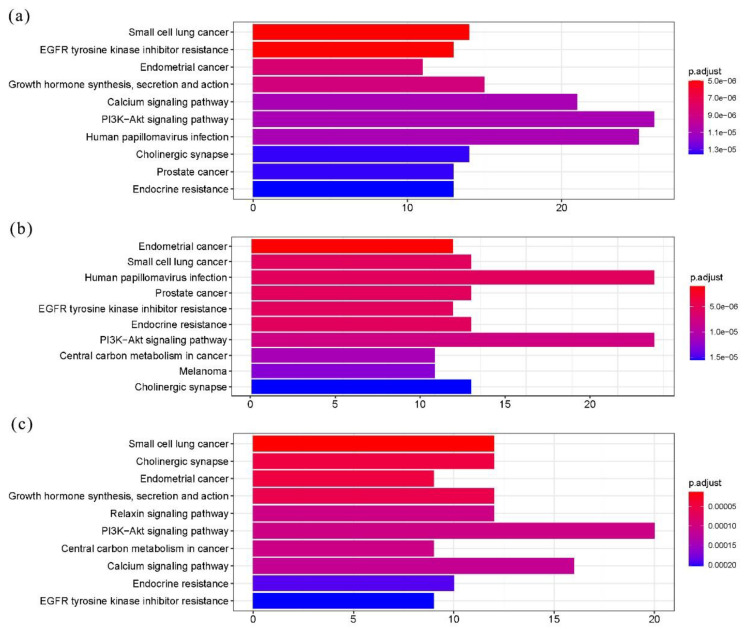
KEGG analyses for mutated genes from three subsets. (**a**) Enriched pathways for overall mutated genes. (**b**) Enriched pathways for baseline mutated genes. (**c**) Enriched pathways for mutated genes after chemotherapy. Top 10 pathways were shown. Length of each column indicates number of enriched genes, and color of bars represents statistical significance.

On KEGG analysis, 96, 97, and 108 pathways were enriched in the baseline, after chemotherapy, and overall subsets, respectively (as illustrated in Table S4). Among the top ten pathways, six were included in all three subsets (i.e., small cell lung cancer, EGFR tyrosine kinase inhibitor resistance, endometrial cancer, PI3K-Akt signaling pathway, endocrine resistance, and cholinergic synapse; as illustrated in Figure 2). Most pathways were typical and indeed meaningful for cancer development, progression, or therapeutic response. The results revealed no significant discrepancies in overall enriched pathways among the three subsets.

### 3.3. Distribution of Gene Mutations and Clinical Characteristics

The patients were divided into different groups according to their clinical characteristics for further analysis. The results show that the ctDNA mutations were significantly associated with tumor size and HER2 status. Patients with tumors > 3 cm carried more mutations than those with tumors ≤ 3 cm (92.29 vs. 66.69, respectively, *p* = 0.035), while HER2(+) patients carried fewer mutations than HER2(−) patients (68.65 vs. 99.20, respectively, *p* = 0.029). For single genes, the mutation profiles of ctDNA also exhibited discrepancies. We selected the top 20 genes for further analysis. The results show that mutations in *AQP7* and *PTEN* were significantly increased in patients with a poor therapeutic effect showing PD (*p* < 0.05). *PIK3CA* mutations occurred more frequently in patients with tumors > 3 cm (*p* = 0.039). The same was also observed for *DNAJC11*, *MAP3K1*, and *PGAP1* in patients with late menarche (≥14 years) and *KRAS* in older patients (age at diagnosis > 51 years, *p* < 0.05) (as illustrated in Table S2).

A multivariate regression analysis was conducted to examine the relations between mutation burden and clinical parameters (as illustrated in Table 2). The results showed that ctDNA mutations were significantly associated with HER2 status. Patients with HER2(+) carried fewer mutations than HER2(−) patients (*OR* 0.02, 95% *CI* 0–0.62, *p* = 0.025). No significant associations were found for other characteristics.

### 3.4. Dynamics of ctDNA Mutations during Chemotherapy

**Figure 3:**
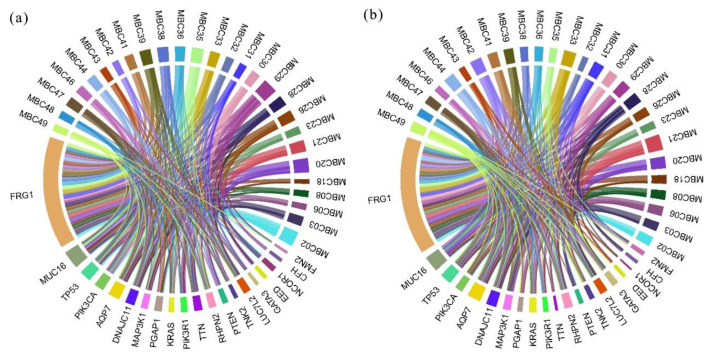
Distribution of ctDNA mutations before and after chemotherapy for all patients. Top 20 genes were shown. (**a**) ctDNA mutations at baseline. (**b**) ctDNA mutations after chemotherapy. Width of each part represents number of mutations detected in corresponding patients or genes.

A total of 768 ctDNA mutations were detected in 302 genes at baseline, which decreased to 633 in 291 genes after three courses of treatment. The mean number of mutations for each patient also decreased after chemotherapy (50.93 vs. 46.59, respectively). To examine the dynamic changes, we compared the distribution of ctDNA mutations in genes before and after chemotherapy (as illustrated in Figure 3). The mutations reduced sharply after chemotherapy for *MUC16*, *NCOR1*, *TTN*, *PIK3R1*, and *TP53*. In contrast, more mutations were detected after chemotherapy for *MYO6*, *FLNC*, *FMN2*, *CDH1*, and *RHO*.

An analysis based on clinical information showed different mutation patterns in plasma DNA between baseline and after chemotherapy (as illustrated in Table S2). For baseline ctDNA, mutations in *PTEN* increased significantly in patients with PD compared to that of patients with PR/SD (*p* = 0.028). More mutations were detected in gene *PIK3CA* in patients with tumors > 3 cm (*p* = 0.039), in *MAP3K1* and *PGAP1* in patients with late menarche (≥14 years, *p* = 0.006 and 0.008, respectively), and in *KRAS* in older patients (age > 51 years, *p* = 0.018). These associations were consistent in all samples, but not in samples obtained after treatment. In addition, fewer ctDNA mutations were found in *AQP7* in PR(+) patients (*p* = 0.023), and in *KRAS* and *PIK3CA* in HER2(+) patients (*p* = 0.041 and 0.030, respectively). In ctDNA after chemotherapy, the number of mutations decreased significantly in *TP53* in both HER2(+) and PR(−) patients (*p* = 0.011 and 0.024, respectively). Mutations in *LUC7L2* were also strongly associated with HER2(−) status (*p* = 0.020).

### 3.5. ctDNA Mutations and Clinical Outcomes

**Figure 4:**
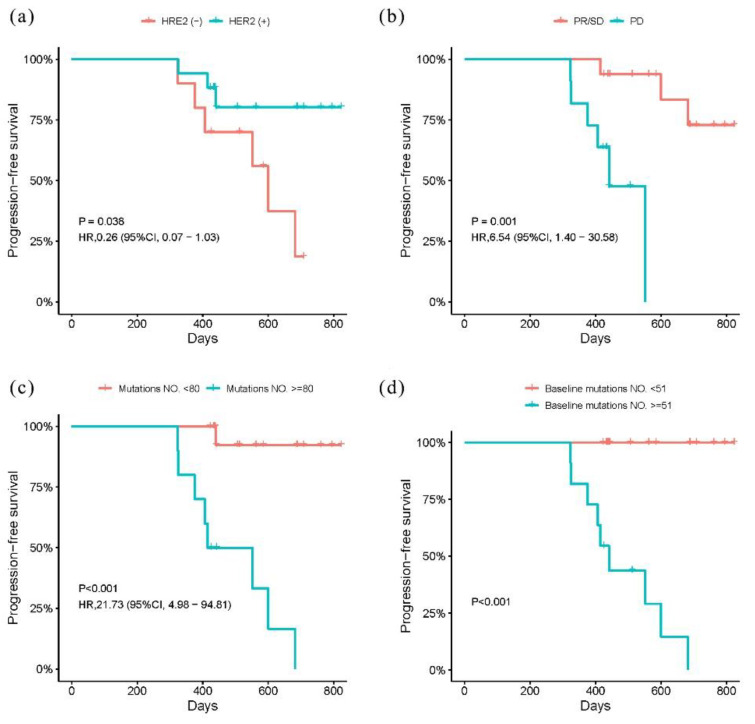
Kaplan–Meier estimate of PFS. (**a**) Kaplan–Meier curves for HER2 status. (**b**) Kaplan–Meier curves for therapeutic effect. (**c**) Kaplan–Meier curves for mutations in overall ctDNA. (**d**) Kaplan–Meier curves for mutations in baseline ctDNA.

A Kaplan–Meier analysis was performed to explore risk factors correlated with PFS, defined as the duration from first sampling to first disease progression. The mean PFS was 537 days (range, 324 to 823 days). All patients were stratified with the mean number of ctDNA mutations at the threshold. The results indicated that HER2 status, therapeutic effect, and number of ctDNA mutations exhibited significant associations with PFS (as illustrated in Figure 4). HER2(+) patients had significantly longer PFS compared with that of HER2(−) patients (adjusted *HR* 0.26, *p* = 0.038), but PD was related to shorter PFS than PR/SD (adjusted *HR* 6.54, *p* = 0.001). Patients carrying more ctDNA mutations overall and in baseline plasma both had poor PFS rates (*p* < 0.001). No associations were found for other clinical characteristics and mutations in ctDNA after chemotherapy.

## 4. Discussion

In clinical practice, the diagnosis and evaluation for breast cancer are based on tissue biopsy with immunohistochemical and cytogenetic tests, but it is invasive and difficult to perform multiple sampling [19,20]. Carcinoembryonic antigen (CEA) and cancer antigen 15-3 (CA15-3) are also used extensively as predictive marker [21]. However, it was demonstrated that only 7.2% and 12.3% of patients exhibited significantly elevated serum CEA and CA15-3 levels, respectively, among Chinese women [22]. Therefore, there is a need for more convenient and specific genetic markers of breast cancer. As ctDNA is double-stranded nucleic acid shed by tumor cells into the circulating blood, it should contain all of the genetic information present in tumor tissue and could capture both spatial and temporal heterogeneity of tumors [23,24]. The application of ctDNA-based real-time liquid biopsy represents a noninvasive and highly sensitive biomarker for cancer diagnosis, early prediction, and therapeutic response assessment [8,25,26,27]. Although there were many studies regarding ctDNA and breast cancer, some challenges and limitations remain for its clinical implementation, including the identification of specific driver mutations for the multiple demands of breast cancer management, the high costs of detecting the low proportion of ctDNA in the “sea” of normal DNA, and the development of standardized methods for data processing [16,28,29]. The development of NGS and bioinformatics provided new opportunities for the establishment and validation of specific panels of mutation biomarkers for routine clinical use in breast cancer management [30,31].

The prevalence of mutations in ctDNA was strikingly similar to that of matched tumor DNA, and they were more often detected in patients with advanced or metastatic cancer [32]. However, most studies to date focused only on a limited repertoire of genes, and they also varied in the quality of samples and sequencing data [33]. In this study, we performed target-capture NGS, which is more sensitive and specific than traditional sequencing, to detect and evaluate the ctDNA in MBC patients. In our cohort, 1182 nonsynonymous mutations in 419 genes were identified. More attention should be paid to some frequently mutated genes, including *FRG1*, *AQP7*, *DNAJC11*, and *MUC16*, in future studies. Mutations were detected in genes closely associated with cancer development, progression, or therapeutic response.

Mutations in ctDNA could reflect intratumoral heterogeneity and disease processes [34,35]. The mutation burden was therefore associated with clinical parameters, representing the phenotype of cancer development [32,36]. We hypothesized that patients with “risk” clinical factors for breast cancer should harbor more mutations [37]. As predicted, our statistical analysis showed that an elevated ctDNA mutation burden was positively correlated with large primary tumor size, HER2(−) status, and poor survival outcome. These were all detrimental to cancer management. In addition, this study suggests that ctDNA mutations could be used as potential biomarkers for the evaluation and prediction of MBC to guide clinical management. Mutations in baseline ctDNA were more effective than those after chemotherapy.

The remarkable advantages of this study are the use of a custom-designed panel with broad coverage, standardized sequencing with high depth, sampling at two time points, and parallel sequencing to eliminate the interference of CH variants. However, this study also has some limitations. First, the sample size was small due to the lack of willingness among MBC patients to supply adequate amounts of blood. Some patients did not agree to participate in genomic testing even though it was provided without cost. Another limitation was the lack of paired tumor tissue samples, which are helpful for verifying the mutations in ctDNA. These limitations restricted further analysis. Despite these limitations, our study contributes to the identification of MBC-related mutations and strengthens the evidence for the clinical applicability of ctDNA detection. Subsequent studies with larger numbers of participants and more complete information are already in progress in our laboratory.

## 5. Conclusions

Mutations in ctDNA were successfully detected in MBC patients by targeted NGS. The mutation burden was significantly associated with clinical factors such as age at diagnosis, tumor size, hormone receptor status, therapeutic effect, and survival. The results suggested that ctDNA could be used as a potential biomarker to evaluate and predict the progression and treatment outcomes of MBC, though more data were needed to optimize the evaluation system.

## Figures and Tables

**Table 1:** Clinical characteristics of MBC patients.

Characteristics		*N* (%)
Diagnostic age (years)	Mean (rang)	51.30 (33–68)
Menarche age (years)	Mean (rang)	14.33 (11–19)
ER status	ER(+)	17 (62.96%)
PR ^a^ status	PR ^a^ (+)	16 (59.26%)
HER2 status	HER2(+)	17 (62.96%)
	* HER2(+) only	7 (25.93%)
Menopause	YES	11 (44.44%)
Size of tumor	>3cm	14 (51.85%)
	≤3cm	13 (48.15%)
T stage	T3	4 (14.81%)
	T4	23 (85.19%)
N stage	N2	6 (22.22%)
	N3	21 (77.78%)
M stage	M1	27 (100.00%)
Metastatic sites	Lymph nodes	24 (88.89%)
	Pulmonary	9 (33.33%)
	Hepatic	14 (51.85%)
	Bone	15 (55.56%)
	Brain	1 (3.70%)
Parturitions	≥3	11 (40.74%)
	<3	16 (59.26%)
Therapeutic effect	PR ^b^/SD	16 (59.26%)
	PD	11 (40.74%)

ER: estrogen receptor; ^a^ PR: progesterone receptor; HER2: human epidermal growth factor receptor 2; ^b^ PR: partial remission; SD: stable disease; PD: progressive disease; * ER(−), PR(−), and HER2(−).

**Table 2:** Multivariate regression analysis of relationship between ctDNA mutations and clinical parameters.

Factors	Coefficient (SE)	Adjusted *OR*	95% *CI*	*p* Value
Diagnostic age	0.12	0.92	0.73–1.16	0.475
Menarche age	0.41	1.57	0.70–3.55	0.274
ER status, ER(+) vs. ER(−)	2.29	2.51	0.03–222.34	0.687
PR ^a^ status, PR ^a^ (+) vs. PR ^a^ (−)	2.07	0.07	0–4.30	0.210
HER2 status, HER2(+) vs. HER2(−)	1.70	0.02	0–0.62	0.025
Menopause, YES vs. NO	2.02	1.48	0.03–77.85	0.847
Size of tumor, >3 cm vs. ≤3 cm	1.55	10.28	0.50–213.38	0.132
Parturitions NO. ≥3 vs. <3	0.50	1.02	0.38–2.72	0.965
Therapeutic effect, PR ^b^/SD vs. PD	1.33	4.26	0.31–58.24	0.277

ER: estrogen receptor; ^a^ PR: progesterone receptor; HER2: human epidermal growth factor receptor 2; ^b^ PR: partial remission; SD: stable disease; PD: progressive disease.

## Data Availability

Data are available to qualified researchers upon reasonable request of the authors.

## Supplementary Materials

The following are available online at https://www.mdpi.com/article/10.3390/curroncol28040214/s1, Figure S1: GO analyses for the mutated genes from three subsets, Table S1: Details of the personalized target-capture sequencing panel, Table S2: Analyses for the distribution of ctDNA mutations for all patients, Table S3: GO analysis for the mutated genes of three subsets, Table S4. KEGG analysis for the mutated genes of three subsets.
