# Supplementary material for: Targeted Next-Generation Sequencing of Circulating Tumor DNA Mutations among Metastatic Breast Cancer Patients
Source: Curr Oncol. 2021 Jun 24;28(4):2326–36. doi: 10.3390/curroncol28040214 (PMC8293138; doi:10.3390/curroncol28040214)
Supplement: Supplementary file 1 [file curroncol-28-00214-s001.zip › curroncol-1215321-supplementary.pdf]

Table S1. Details of the personalized target-capture sequencing panel.

Chromosomes	Genes	Exons	Length (bp)
chr.1	98	109	14226
chr.2	74	96	11947
chr.3	45	54	8172
chr.4	29	32	3099
chr.5	42	54	7141
chr.6	33	35	3932
chr.7	34	37	3809
chr.8	30	31	3080
chr.9	33	36	3189
chr.10	27	33	5001
chr.11	41	43	3939
chr.12	48	50	5587
chr.13	13	14	1447
chr.14	21	21	2012
chr.15	26	27	2246
chr.16	24	34	4130
chr.17	51	67	6819
chr.18	15	15	1540
chr.19	60	65	16244
chr.20	24	28	2853
chr.21	5	8	941
chr.22	14	14	1226
chr.X	48	58	6618
Total	835	961	119198

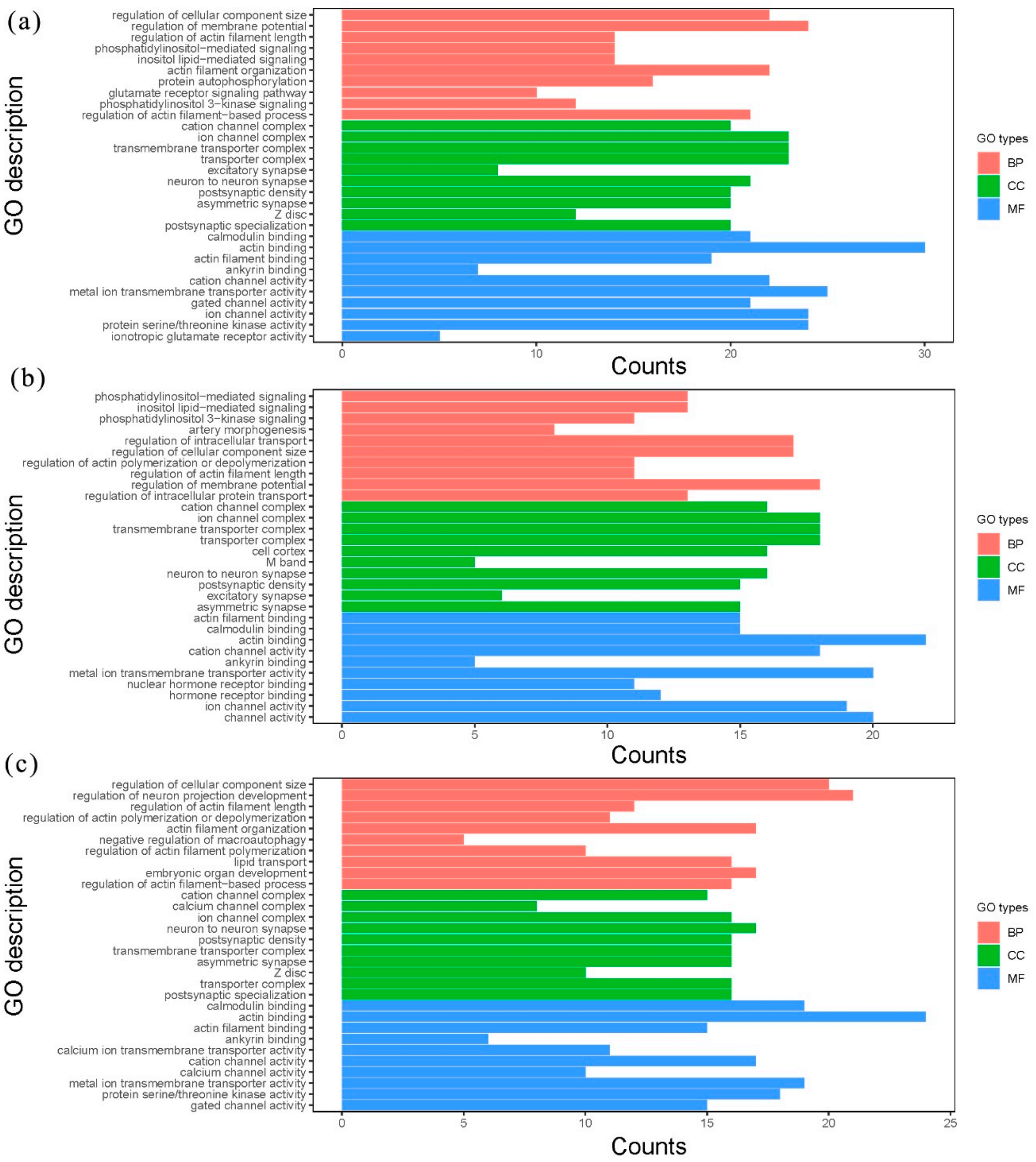

Figure S1. GO analyses for the mutated genes from three subsets. (a) GO analysis for overall mutated genes. (b) GO analysis for baseline mutated genes. (c) GO analysis for mutated genes from after chemotherapy. The length of each column indicates the number of enriched genes, which ranked according to statistical significance.

Table S2. Analyses for the distribution of ctDNA mutations for all patients

clinical characteristics	All genes			*AQP7*			*KRAS*			*MAP3K1*			*PGAP1*		
	**All**	**Before**	**After**	**All**	**Before**	**After**	**All**	**Before**	**After**	**All**	**Before**	**After**	**All**	**Before**	**After**
Diagnostic age, > 51 vs ≤51	0.269	0.139	0.954	0.335	0.249	0.650	0.039	0.018	0.363	0.759	0.495	0.959	0.291	0.766	0.085
Menarche age, ≥14 vs < 14	0.355	0.722	0.044	0.320	0.555	0.272	0.289	0.953	0.354	0.031	0.006	0.201	0.003	0.008	0.109
ER status, ER(+) vs ER(-)	0.304	0.235	0.867	0.880	0.395	0.635	0.557	0.230	0.677	0.822	0.380	0.740	0.720	0.393	0.699
PR[a] status, PR[a] (+) vs PR[a] (-)	0.319	0.241	0.989	0.202	0.023	0.318	0.525	0.789	0.384	0.896	0.693	0.584	0.523	0.460	0.889
HER2 status, HER2(+) vs HER2(-)	0.029	0.022	0.639	0.389	0.432	0.223	0.469	0.041	0.439	0.968	0.826	0.966	0.083	0.130	0.257
Size of tumor, ＞3cm vs ≤3cm	0.035	0.047	0.196	0.750	0.712	0.666	0.279	0.135	0.675	0.247	0.090	0.689	0.725	0.887	0.128
Therapeutic effect, PR[b]/SD vs PD	0.178	0.045	0.919	0.000	0.082	0.003	0.091	0.401	0.305	0.379	0.957	0.250	0.201	0.154	0.889
Parturitions NO. ≥3 vs ＜3	0.684	0.551	0.968	0.746	0.190	0.852	0.497	0.435	0.124	0.701	0.237	0.818	0.796	0.804	0.889
Menopause, YES vs NO	0.422	0.285	0.877	0.341	0.090	0.897	0.393	0.610	0.349	0.822	0.700	0.954	0.807	0.771	0.275

clinical characteristics	*LUC7L2*			*DNAJC11*			*PIK3CA*			*PTEN*			*TP53*		
	**All**	**Before**	**After**	**All**	**Before**	**After**	**All**	**Before**	**After**	**All**	**Before**	**After**	**All**	**Before**	**After**
Diagnostic age, > 51 vs ≤51	0.454	0.472	0.832	0.095	0.334	0.082	0.188	0.236	0.564	0.175	0.401	0.305	0.138	0.170	0.970
Menarche age, ≥14 vs < 14	0.454	0.109	0.832	0.019	0.334	0.689	0.848	0.474	0.096	1.000	0.500	0.569	0.074	0.453	0.068
ER status, ER(+) vs ER(-)	0.081	0.074	0.074	0.716	0.332	0.083	0.382	0.005	0.144	1.000	0.703	0.580	0.768	0.760	0.166
PR[a] status, PR[a] (+) vs PR[a] (-)	0.397	0.382	0.382	0.944	0.333	0.083	0.449	0.194	0.722	0.814	0.542	0.450	0.305	0.494	0.024
HER2 status, HER2(+) vs HER2(-)	0.083	0.363	0.020	0.795	0.332	0.351	0.104	0.030	0.647	0.100	0.180	0.243	0.677	0.449	0.011
Size of tumor, ＞3cm vs ≤3cm	0.599	0.577	0.170	0.182	0.336	0.082	0.039	0.039	0.389	1.000	0.304	0.205	0.340	0.048	0.380
Therapeutic effect, PR[b]/SD vs PD	0.397	0.972	0.972	0.176	0.333	0.787	0.112	0.337	0.344	0.027	0.028	0.140	0.967	0.220	0.067
Parturitions NO. ≥3 vs ＜3	0.787	0.382	0.280	0.517	0.333	0.397	0.785	0.406	0.020	0.426	0.868	0.061	0.837	0.822	0.770
Menopause, YES vs NO	0.689	0.832	0.472	0.432	0.339	0.454	0.786	0.774	0.942	0.114	0.318	0.179	0.066	0.164	0.771

P value were shown in the table. All: analyses for all samples; Before: analyses for baseline samples. After: analyses for samples after three courses of treatment. Only mutated genes with statistical significance were shown. ER: estrogen receptor; [a]PR: progesterone receptor; HER2: human epidermal growth factor receptor 2; [b]PR: partial remission; SD: stable disease; PD: progressive disease

Table S3. GO analysis for the mutated genes of three subsets

ID	Subjects correlated with BP for all mutated genes (53)	Adjusted *P* value
GO:0032535	regulation of cellular component size	0.018
GO:0042391	regulation of membrane potential	0.018
GO:0030832	regulation of actin filament length	0.018
GO:0048015	phosphatidylinositol-mediated signaling	0.018
GO:0048017	inositol lipid-mediated signaling	0.018
GO:0007015	actin filament organization	0.018
GO:0046777	protein autophosphorylation	0.018
GO:0007215	glutamate receptor signaling pathway	0.020
GO:0014065	phosphatidylinositol 3-kinase signaling	0.023
GO:0032970	regulation of actin filament-based process	0.023
GO:0018108	peptidyl-tyrosine phosphorylation	0.025
GO:0018212	peptidyl-tyrosine modification	0.026
GO:0008064	regulation of actin polymerization or depolymerization	0.026
GO:0051899	membrane depolarization	0.026
GO:0010975	regulation of neuron projection development	0.026
GO:0050805	negative regulation of synaptic transmission	0.026
GO:0030239	myofibril assembly	0.026
GO:0035249	synaptic transmission, glutamatergic	0.026
GO:0051146	striated muscle cell differentiation	0.026
GO:0030833	regulation of actin filament polymerization	0.026
GO:0035235	ionotropic glutamate receptor signaling pathway	0.026
GO:1902903	regulation of supramolecular fiber organization	0.026
GO:0043242	negative regulation of protein-containing complex disassembly	0.027
GO:0048844	artery morphogenesis	0.027
GO:0055003	cardiac myofibril assembly	0.027
GO:0034765	regulation of ion transmembrane transport	0.028
GO:0006936	muscle contraction	0.030
GO:0051494	negative regulation of cytoskeleton organization	0.035
GO:0055002	striated muscle cell development	0.035
GO:0048706	embryonic skeletal system development	0.037
GO:0032956	regulation of actin cytoskeleton organization	0.037
GO:0048701	embryonic cranial skeleton morphogenesis	0.037
GO:1901879	regulation of protein depolymerization	0.037
GO:0006978	DNA damage response, signal transduction by p53 class mediator resulting in transcription of p21 class mediator	0.037
GO:0031000	response to caffeine	0.037
GO:0036270	response to diuretic	0.037
GO:0110053	regulation of actin filament organization	0.037
GO:0048738	cardiac muscle tissue development	0.037
GO:0060292	long-term synaptic depression	0.038
GO:0030041	actin filament polymerization	0.038
GO:0008154	actin polymerization or depolymerization	0.038
GO:1901880	negative regulation of protein depolymerization	0.038

GO:1904888	cranial skeletal system development	0.038
GO:0042692	muscle cell differentiation	0.038
GO:0051261	protein depolymerization	0.038
GO:0042772	DNA damage response, signal transduction resulting in transcription	0.038
GO:0055001	muscle cell development	0.042
GO:0055013	cardiac muscle cell development	0.042
GO:0016242	negative regulation of macroautophagy	0.044
GO:0043244	regulation of protein-containing complex disassembly	0.045
GO:0048568	embryonic organ development	0.047
GO:0006814	sodium ion transport	0.047
GO:0010927	cellular component assembly involved in morphogenesis	0.048
**ID**	**Subjects correlated with CC for all mutated genes (43)**	**Adjusted *P* value**
GO:0034703	cation channel complex	0.000
GO:0034702	ion channel complex	0.000
GO:1902495	transmembrane transporter complex	0.000
GO:1990351	transporter complex	0.000
GO:0060076	excitatory synapse	0.001
GO:0098984	neuron to neuron synapse	0.001
GO:0014069	postsynaptic density	0.001
GO:0032279	asymmetric synapse	0.001
GO:0030018	Z disc	0.001
GO:0099572	postsynaptic specialization	0.001
GO:0031674	I band	0.001
GO:0034704	calcium channel complex	0.002
GO:0008328	ionotropic glutamate receptor complex	0.002
GO:0034706	sodium channel complex	0.003
GO:0098878	neurotransmitter receptor complex	0.003
GO:0005938	cell cortex	0.005
GO:0030016	myofibril	0.005
GO:0005925	focal adhesion	0.005
GO:0016528	sarcoplasm	0.005
GO:0031430	M band	0.005
GO:0030017	sarcomere	0.005
GO:0098688	parallel fiber to Purkinje cell synapse	0.005
GO:0005875	microtubule associated complex	0.005
GO:0030055	cell-substrate junction	0.005
GO:0043292	contractile fiber	0.005
GO:0031252	cell leading edge	0.009
GO:0098686	hippocampal mossy fiber to CA3 synapse	0.009
GO:0098793	presynapse	0.015
GO:0031672	A band	0.016
GO:0033017	sarcoplasmic reticulum membrane	0.016
GO:0043204	perikaryon	0.023
GO:0098978	glutamatergic synapse	0.027

GO:0030864	cortical actin cytoskeleton	0.030
GO:0045211	postsynaptic membrane	0.030
GO:0031256	leading edge membrane	0.030
GO:0030863	cortical cytoskeleton	0.030
GO:0001518	voltage-gated sodium channel complex	0.032
GO:0097060	synaptic membrane	0.034
GO:0005604	basement membrane	0.034
GO:0016529	sarcoplasmic reticulum	0.034
GO:0033116	endoplasmic reticulum-Golgi intermediate compartment membrane	0.034
GO:0062023	collagen-containing extracellular matrix	0.038
GO:0070603	SWI/SNF superfamily-type complex	0.046
**ID**	**Subjects correlated with MF for all mutated genes (28)**	**Adjusted *P* value**
GO:0005516	calmodulin binding	0.000
GO:0003779	actin binding	0.000
GO:0051015	actin filament binding	0.000
GO:0030506	ankyrin binding	0.000
GO:0005261	cation channel activity	0.001
GO:0046873	metal ion transmembrane transporter activity	0.001
GO:0022836	gated channel activity	0.002
GO:0005216	ion channel activity	0.002
GO:0004674	protein serine/threonine kinase activity	0.002
GO:0004970	ionotropic glutamate receptor activity	0.002
GO:0015267	channel activity	0.002
GO:0022803	passive transmembrane transporter activity	0.002
GO:0015085	calcium ion transmembrane transporter activity	0.002
GO:0051427	hormone receptor binding	0.002
GO:0008307	structural constituent of muscle	0.002
GO:0005200	structural constituent of cytoskeleton	0.003
GO:0005262	calcium channel activity	0.003
GO:0035257	nuclear hormone receptor binding	0.004
GO:0008066	glutamate receptor activity	0.009
GO:0061629	RNA polymerase II-specific DNA-binding transcription factor binding	0.013
GO:0030165	PDZ domain binding	0.017
GO:0004713	protein tyrosine kinase activity	0.021
GO:0019838	growth factor binding	0.023
GO:0005201	extracellular matrix structural constituent	0.024
GO:0008331	high voltage-gated calcium channel activity	0.028
GO:0140297	DNA-binding transcription factor binding	0.043
GO:0005272	sodium channel activity	0.043
GO:0030020	extracellular matrix structural constituent conferring tensile strength	0.043
**ID**	**Subjects correlated with BP for baseline (42)**	**Adjusted *P* value**
GO:0048015	phosphatidylinositol-mediated signaling	0.007

GO:0048017	inositol lipid-mediated signaling	0.007
GO:0014065	phosphatidylinositol 3-kinase signaling	0.015
GO:0048844	artery morphogenesis	0.015
GO:0032386	regulation of intracellular transport	0.023
GO:0032535	regulation of cellular component size	0.023
GO:0008064	regulation of actin polymerization or depolymerization	0.025
GO:0030832	regulation of actin filament length	0.025
GO:0042391	regulation of membrane potential	0.025
GO:0033157	regulation of intracellular protein transport	0.025
GO:0006978	DNA damage response, signal transduction by p53 class mediator resulting in transcription of p21 class mediator	0.025
GO:0007015	actin filament organization	0.025
GO:0006814	sodium ion transport	0.025
GO:0034765	regulation of ion transmembrane transport	0.025
GO:0016242	negative regulation of macroautophagy	0.025
GO:0060840	artery development	0.025
GO:0042772	DNA damage response, signal transduction resulting in transcription	0.025
GO:0051146	striated muscle cell differentiation	0.025
GO:0110053	regulation of actin filament organization	0.027
GO:0030833	regulation of actin filament polymerization	0.027
GO:2000310	regulation of NMDA receptor activity	0.030
GO:0032956	regulation of actin cytoskeleton organization	0.031
GO:0032970	regulation of actin filament-based process	0.035
GO:1902903	regulation of supramolecular fiber organization	0.038
GO:0008154	actin polymerization or depolymerization	0.039
GO:0032008	positive regulation of TOR signaling	0.039
GO:0043010	camera-type eye development	0.039
GO:0018108	peptidyl-tyrosine phosphorylation	0.041
GO:0055013	cardiac muscle cell development	0.041
GO:0097306	cellular response to alcohol	0.041
GO:1900449	regulation of glutamate receptor signaling pathway	0.041
GO:0018212	peptidyl-tyrosine modification	0.041
GO:0030041	actin filament polymerization	0.041
GO:0060537	muscle tissue development	0.041
GO:0051899	membrane depolarization	0.041
GO:0035249	synaptic transmission, glutamatergic	0.046
GO:0030241	skeletal muscle myosin thick filament assembly	0.046
GO:0071688	striated muscle myosin thick filament assembly	0.046
GO:0072659	protein localization to plasma membrane	0.046
GO:0055006	cardiac cell development	0.046
GO:0031929	TOR signaling	0.046
GO:0055003	cardiac myofibril assembly	0.046
**ID**	**Subjects correlated with CC for baseline (33)**	**Adjusted *P* value**
GO:0034703	cation channel complex	0.000

GO:0034702	ion channel complex	0.000
GO:1902495	transmembrane transporter complex	0.000
GO:1990351	transporter complex	0.000
GO:0005938	cell cortex	0.001
GO:0031430	M band	0.003
GO:0098984	neuron to neuron synapse	0.003
GO:0014069	postsynaptic density	0.003
GO:0060076	excitatory synapse	0.003
GO:0032279	asymmetric synapse	0.003
GO:0099572	postsynaptic specialization	0.006
GO:0031672	A band	0.008
GO:0098793	presynapse	0.008
GO:0030863	cortical cytoskeleton	0.008
GO:0034706	sodium channel complex	0.008
GO:0030864	cortical actin cytoskeleton	0.008
GO:0030018	Z disc	0.015
GO:0031256	leading edge membrane	0.018
GO:0008328	ionotropic glutamate receptor complex	0.018
GO:0098878	neurotransmitter receptor complex	0.019
GO:0001518	voltage-gated sodium channel complex	0.019
GO:0031674	I band	0.020
GO:0098686	hippocampal mossy fiber to CA3 synapse	0.024
GO:0097060	synaptic membrane	0.024
GO:0098688	parallel fiber to Purkinje cell synapse	0.024
GO:0031252	cell leading edge	0.033
GO:0042734	presynaptic membrane	0.035
GO:0045211	postsynaptic membrane	0.035
GO:0016514	SWI/SNF complex	0.035
GO:0034704	calcium channel complex	0.035
GO:0031253	cell projection membrane	0.035
GO:0030017	sarcomere	0.036
GO:0031258	lamellipodium membrane	0.048
**ID**	**Subjects correlated with MF for baseline (32)**	**Adjusted *P* value**
GO:0051015	actin filament binding	0.000
GO:0005516	calmodulin binding	0.000
GO:0003779	actin binding	0.000
GO:0005261	cation channel activity	0.001
GO:0030506	ankyrin binding	0.001
GO:0046873	metal ion transmembrane transporter activity	0.002
GO:0035257	nuclear hormone receptor binding	0.002
GO:0051427	hormone receptor binding	0.002
GO:0005216	ion channel activity	0.003
GO:0015267	channel activity	0.003
GO:0022803	passive transmembrane transporter activity	0.003
GO:0022836	gated channel activity	0.004

GO:0061629	RNA polymerase II-specific DNA-binding transcription factor binding	0.005
GO:0005200	structural constituent of cytoskeleton	0.007
GO:0140297	DNA-binding transcription factor binding	0.015
GO:0008331	high voltage-gated calcium channel activity	0.015
GO:0004674	protein serine/threonine kinase activity	0.017
GO:0005262	calcium channel activity	0.020
GO:0008307	structural constituent of muscle	0.020
GO:0042805	actinin binding	0.020
GO:0005201	extracellular matrix structural constituent	0.025
GO:0005244	voltage-gated ion channel activity	0.025
GO:0022832	voltage-gated channel activity	0.025
GO:0019838	growth factor binding	0.031
GO:0015085	calcium ion transmembrane transporter activity	0.033
GO:0016922	nuclear receptor binding	0.035
GO:0032794	GTPase activating protein binding	0.037
GO:0003777	microtubule motor activity	0.039
GO:0043178	alcohol binding	0.040
GO:0051393	alpha-actinin binding	0.046
GO:0008569	ATP-dependent microtubule motor activity, minus-end-directed	0.046
GO:0005543	phospholipid binding	0.049
**ID**	**Subjects correlated with BP after chemotherapy (33)**	**Adjusted *P* value**
GO:0032535	regulation of cellular component size	0.001
GO:0010975	regulation of neuron projection development	0.013
GO:0030832	regulation of actin filament length	0.013
GO:0008064	regulation of actin polymerization or depolymerization	0.046
GO:0007015	actin filament organization	0.046
GO:0016242	negative regulation of macroautophagy	0.049
GO:0030833	regulation of actin filament polymerization	0.049
GO:0006869	lipid transport	0.049
GO:0048568	embryonic organ development	0.049
GO:0032970	regulation of actin filament-based process	0.049
GO:0042391	regulation of membrane potential	0.049
GO:0008154	actin polymerization or depolymerization	0.049
GO:0010038	response to metal ion	0.049
GO:0006936	muscle contraction	0.049
GO:0051592	response to calcium ion	0.049
GO:0048015	phosphatidylinositol-mediated signaling	0.049
GO:0030041	actin filament polymerization	0.049
GO:0010528	regulation of transposition	0.049
GO:0010529	negative regulation of transposition	0.049
GO:0010876	lipid localization	0.049
GO:0048017	inositol lipid-mediated signaling	0.049
GO:0051899	membrane depolarization	0.049
GO:0071313	cellular response to caffeine	0.049

GO:0001704	formation of primary germ layer	0.049
GO:0010881	regulation of cardiac muscle contraction by regulation of the release of sequestered calcium ion	0.049
GO:1904888	cranial skeletal system development	0.049
GO:0048704	embryonic skeletal system morphogenesis	0.049
GO:0110053	regulation of actin filament organization	0.049
GO:0031929	TOR signaling	0.049
GO:0048701	embryonic cranial skeleton morphogenesis	0.049
GO:0086016	AV node cell action potential	0.049
GO:0086027	AV node cell to bundle of His cell signaling	0.049
GO:0048010	vascular endothelial growth factor receptor signaling pathway	0.049
**ID**	**Subjects correlated with CC after chemotherapy (38)**	**Adjusted *P* value**
GO:0034703	cation channel complex	0.000
GO:0034704	calcium channel complex	0.001
GO:0034702	ion channel complex	0.001
GO:0098984	neuron to neuron synapse	0.001
GO:0014069	postsynaptic density	0.001
GO:1902495	transmembrane transporter complex	0.001
GO:0032279	asymmetric synapse	0.001
GO:0030018	Z disc	0.001
GO:1990351	transporter complex	0.001
GO:0099572	postsynaptic specialization	0.001
GO:0031674	I band	0.001
GO:0031252	cell leading edge	0.002
GO:0030016	myofibril	0.002
GO:0098686	hippocampal mossy fiber to CA3 synapse	0.003
GO:0043292	contractile fiber	0.003
GO:0030017	sarcomere	0.004
GO:0034706	sodium channel complex	0.007
GO:0030027	lamellipodium	0.009
GO:0043204	perikaryon	0.013
GO:0016528	sarcoplasm	0.019
GO:0098978	glutamatergic synapse	0.025
GO:0005938	cell cortex	0.025
GO:0005875	microtubule associated complex	0.025
GO:0043025	neuronal cell body	0.028
GO:0005925	focal adhesion	0.028
GO:0042734	presynaptic membrane	0.030
GO:0030055	cell-substrate junction	0.030
GO:0045211	postsynaptic membrane	0.030
GO:0033017	sarcoplasmic reticulum membrane	0.031
GO:0098858	actin-based cell projection	0.037
GO:0031256	leading edge membrane	0.037
GO:0000118	histone deacetylase complex	0.037
GO:0097060	synaptic membrane	0.037

GO:0042383	sarcolemma	0.037
GO:0005891	voltage-gated calcium channel complex	0.037
GO:0005770	late endosome	0.038
GO:0031258	lamellipodium membrane	0.040
GO:0070603	SWI/SNF superfamily-type complex	0.047
**ID**	**Subjects correlated with MF after chemotherapy (20)**	**Adjusted *P* value**
GO:0005516	calmodulin binding	0.000
GO:0003779	actin binding	0.000
GO:0051015	actin filament binding	0.000
GO:0030506	ankyrin binding	0.000
GO:0015085	calcium ion transmembrane transporter activity	0.001
GO:0005261	cation channel activity	0.001
GO:0005262	calcium channel activity	0.001
GO:0046873	metal ion transmembrane transporter activity	0.003
GO:0004674	protein serine/threonine kinase activity	0.008
GO:0022836	gated channel activity	0.013
GO:0015267	channel activity	0.015
GO:0022803	passive transmembrane transporter activity	0.015
GO:0005216	ion channel activity	0.015
GO:0008331	high voltage-gated calcium channel activity	0.015
GO:0004712	protein serine/threonine/tyrosine kinase activity	0.017
GO:0008307	structural constituent of muscle	0.022
GO:0005245	voltage-gated calcium channel activity	0.025
GO:0005200	structural constituent of cytoskeleton	0.028
GO:0031432	titin binding	0.032
GO:0032794	GTPase activating protein binding	0.046

BP: biological process; CC: cellular component; MF: molecular function.

Subjects were ranked according to statistical significance.

Table S4. KEGG analysis for the mutated genes of three subsets

ID	Pathways for all mutated genes (108)	Adjusted *P* value
hsa05222	Small cell lung cancer	0.000
hsa01521	EGFR tyrosine kinase inhibitor resistance	0.000
hsa05213	Endometrial cancer	0.000
hsa04935	Growth hormone synthesis, secretion and action	0.000
hsa04020	Calcium signaling pathway	0.000
hsa04151	PI3K-Akt signaling pathway	0.000
hsa05165	Human papillomavirus infection	0.000
hsa04725	Cholinergic synapse	0.000
hsa05215	Prostate cancer	0.000
hsa01522	Endocrine resistance	0.000
hsa05230	Central carbon metabolism in cancer	0.000
hsa05223	Non-small cell lung cancer	0.000
hsa04510	Focal adhesion	0.000
hsa05214	Glioma	0.000
hsa05212	Pancreatic cancer	0.000
hsa04926	Relaxin signaling pathway	0.000
hsa04724	Glutamatergic synapse	0.000
hsa04010	MAPK signaling pathway	0.000
hsa05163	Human cytomegalovirus infection	0.000
hsa05218	Melanoma	0.000
hsa04728	Dopaminergic synapse	0.000
hsa04713	Circadian entrainment	0.000
hsa04929	GnRH secretion	0.000
hsa04371	Apelin signaling pathway	0.000
hsa05221	Acute myeloid leukemia	0.000
hsa05161	Hepatitis B	0.000
hsa04012	ErbB signaling pathway	0.000
hsa04015	Rap1 signaling pathway	0.000
hsa05224	Breast cancer	0.000
hsa05226	Gastric cancer	0.000
hsa04912	GnRH signaling pathway	0.001
hsa05220	Chronic myeloid leukemia	0.001
hsa04730	Long-term depression	0.001
hsa04930	Type II diabetes mellitus	0.001
hsa05231	Choline metabolism in cancer	0.001
hsa05205	Proteoglycans in cancer	0.001
hsa05146	Amoebiasis	0.001
hsa04720	Long-term potentiation	0.001
hsa05235	PD-L1 expression and PD-1 checkpoint pathway in cancer	0.002
hsa04066	HIF-1 signaling pathway	0.002
hsa01524	Platinum drug resistance	0.002
hsa04970	Salivary secretion	0.002
hsa04923	Regulation of lipolysis in adipocytes	0.002

hsa04210	Apoptosis	0.002
hsa04630	JAK-STAT signaling pathway	0.003
hsa04722	Neurotrophin signaling pathway	0.003
hsa05017	Spinocerebellar ataxia	0.003
hsa04919	Thyroid hormone signaling pathway	0.003
hsa04933	AGE-RAGE signaling pathway in diabetic complications	0.003
hsa04213	Longevity regulating pathway - multiple species	0.003
hsa05225	Hepatocellular carcinoma	0.004
hsa05167	Kaposi sarcoma-associated herpesvirus infection	0.004
hsa04972	Pancreatic secretion	0.004
hsa04611	Platelet activation	0.004
hsa04072	Phospholipase D signaling pathway	0.004
hsa04723	Retrograde endocannabinoid signaling	0.004
hsa04261	Adrenergic signaling in cardiomyocytes	0.004
hsa05210	Colorectal cancer	0.005
hsa04921	Oxytocin signaling pathway	0.005
hsa04664	Fc epsilon RI signaling pathway	0.005
hsa04540	Gap junction	0.005
hsa04211	Longevity regulating pathway	0.005
hsa04014	Ras signaling pathway	0.005
hsa05160	Hepatitis C	0.005
hsa04917	Prolactin signaling pathway	0.006
hsa04140	Autophagy - animal	0.006
hsa04915	Estrogen signaling pathway	0.007
hsa05418	Fluid shear stress and atherosclerosis	0.007
hsa04071	Sphingolipid signaling pathway	0.008
hsa04062	Chemokine signaling pathway	0.008
hsa04810	Regulation of actin cytoskeleton	0.008
hsa04750	Inflammatory mediator regulation of TRP channels	0.009
hsa05100	Bacterial invasion of epithelial cells	0.009
hsa05033	Nicotine addiction	0.009
hsa05219	Bladder cancer	0.010
hsa05142	Chagas disease	0.010
hsa04662	B cell receptor signaling pathway	0.012
hsa04150	mTOR signaling pathway	0.013
hsa04068	FoxO signaling pathway	0.014
hsa04931	Insulin resistance	0.014
hsa04911	Insulin secretion	0.014
hsa04512	ECM-receptor interaction	0.016
hsa04973	Carbohydrate digestion and absorption	0.016
hsa05135	Yersinia infection	0.017
hsa04024	cAMP signaling pathway	0.017
hsa05211	Renal cell carcinoma	0.018
hsa05131	Shigellosis	0.018
hsa04726	Serotonergic synapse	0.019

hsa05166	Human T-cell leukemia virus 1 infection	0.019
hsa04550	Signaling pathways regulating pluripotency of stem cells	0.021
hsa05414	Dilated cardiomyopathy	0.023
hsa04666	Fc gamma R-mediated phagocytosis	0.024
hsa04925	Aldosterone synthesis and secretion	0.025
hsa04971	Gastric acid secretion	0.027
hsa05412	Arrhythmogenic right ventricular cardiomyopathy	0.028
hsa05216	Thyroid cancer	0.031
hsa04625	C-type lectin receptor signaling pathway	0.033
hsa04218	Cellular senescence	0.034
hsa04370	VEGF signaling pathway	0.035
hsa04922	Glucagon signaling pathway	0.037
hsa04270	Vascular smooth muscle contraction	0.038
hsa05132	Salmonella infection	0.042
hsa04742	Taste transduction	0.043
hsa04910	Insulin signaling pathway	0.043
hsa05145	Toxoplasmosis	0.045
hsa05162	Measles	0.046
hsa04927	Cortisol synthesis and secretion	0.048
hsa04727	GABAergic synapse	0.048
**ID**	**Pathways for baseline mutated genes (96)**	**Adjusted *P* value**
hsa05213	Endometrial cancer	0.000
hsa05222	Small cell lung cancer	0.000
hsa05165	Human papillomavirus infection	0.000
hsa05215	Prostate cancer	0.000
hsa01521	EGFR tyrosine kinase inhibitor resistance	0.000
hsa01522	Endocrine resistance	0.000
hsa04151	PI3K-Akt signaling pathway	0.000
hsa05230	Central carbon metabolism in cancer	0.000
hsa05218	Melanoma	0.000
hsa04725	Cholinergic synapse	0.000
hsa05214	Glioma	0.000
hsa05212	Pancreatic cancer	0.000
hsa04935	Growth hormone synthesis, secretion and action	0.000
hsa04929	GnRH secretion	0.000
hsa05224	Breast cancer	0.000
hsa04510	Focal adhesion	0.000
hsa05223	Non-small cell lung cancer	0.000
hsa05220	Chronic myeloid leukemia	0.000
hsa05226	Gastric cancer	0.000
hsa04010	MAPK signaling pathway	0.000
hsa04926	Relaxin signaling pathway	0.000
hsa04012	ErbB signaling pathway	0.000
hsa05221	Acute myeloid leukemia	0.000
hsa05161	Hepatitis B	0.000

hsa04371	Apelin signaling pathway	0.000
hsa05225	Hepatocellular carcinoma	0.000
hsa04919	Thyroid hormone signaling pathway	0.000
hsa05231	Choline metabolism in cancer	0.000
hsa04213	Longevity regulating pathway - multiple species	0.001
hsa04728	Dopaminergic synapse	0.001
hsa05210	Colorectal cancer	0.001
hsa04210	Apoptosis	0.001
hsa04930	Type II diabetes mellitus	0.001
hsa05163	Human cytomegalovirus infection	0.001
hsa04211	Longevity regulating pathway	0.001
hsa04720	Long-term potentiation	0.001
hsa04724	Glutamatergic synapse	0.001
hsa04664	Fc epsilon RI signaling pathway	0.001
hsa05017	Spinocerebellar ataxia	0.001
hsa04912	GnRH signaling pathway	0.001
hsa04071	Sphingolipid signaling pathway	0.001
hsa04722	Neurotrophin signaling pathway	0.001
hsa01524	Platinum drug resistance	0.002
hsa04020	Calcium signaling pathway	0.002
hsa04015	Rap1 signaling pathway	0.002
hsa05146	Amoebiasis	0.002
hsa04923	Regulation of lipolysis in adipocytes	0.003
hsa05166	Human T-cell leukemia virus 1 infection	0.003
hsa04630	JAK-STAT signaling pathway	0.003
hsa04662	B cell receptor signaling pathway	0.003
hsa05167	Kaposi sarcoma-associated herpesvirus infection	0.003
hsa04730	Long-term depression	0.003
hsa05033	Nicotine addiction	0.003
hsa04066	HIF-1 signaling pathway	0.003
hsa04140	Autophagy - animal	0.003
hsa05219	Bladder cancer	0.003
hsa04915	Estrogen signaling pathway	0.003
hsa05418	Fluid shear stress and atherosclerosis	0.003
hsa04540	Gap junction	0.004
hsa05235	PD-L1 expression and PD-1 checkpoint pathway in cancer	0.004
hsa05205	Proteoglycans in cancer	0.004
hsa04072	Phospholipase D signaling pathway	0.005
hsa04723	Retrograde endocannabinoid signaling	0.005
hsa05211	Renal cell carcinoma	0.005
hsa04973	Carbohydrate digestion and absorption	0.005
hsa04917	Prolactin signaling pathway	0.005
hsa04611	Platelet activation	0.006
hsa04713	Circadian entrainment	0.006
hsa04150	mTOR signaling pathway	0.006

hsa04218	Cellular senescence	0.006
hsa05160	Hepatitis C	0.006
hsa04933	AGE-RAGE signaling pathway in diabetic complications	0.007
hsa05142	Chagas disease	0.007
hsa04014	Ras signaling pathway	0.009
hsa04910	Insulin signaling pathway	0.010
hsa04931	Insulin resistance	0.010
hsa04370	VEGF signaling pathway	0.012
hsa05216	Thyroid cancer	0.013
hsa05131	Shigellosis	0.013
hsa04512	ECM-receptor interaction	0.014
hsa04970	Salivary secretion	0.019
hsa04062	Chemokine signaling pathway	0.021
hsa04666	Fc gamma R-mediated phagocytosis	0.022
hsa04750	Inflammatory mediator regulation of TRP channels	0.023
hsa04914	Progesterone-mediated oocyte maturation	0.025
hsa04068	FoxO signaling pathway	0.025
hsa04625	C-type lectin receptor signaling pathway	0.029
hsa04660	T cell receptor signaling pathway	0.029
hsa05100	Bacterial invasion of epithelial cells	0.033
hsa05412	Arrhythmogenic right ventricular cardiomyopathy	0.033
hsa04550	Signaling pathways regulating pluripotency of stem cells	0.037
hsa05145	Toxoplasmosis	0.039
hsa04810	Regulation of actin cytoskeleton	0.041
hsa04726	Serotonergic synapse	0.043
hsa04261	Adrenergic signaling in cardiomyocytes	0.045
hsa04742	Taste transduction	0.048
**ID**	**Pathways for mutated genes after chemotherapy (97)**	**Adjusted *P* value**
hsa05222	Small cell lung cancer	0.000
hsa04725	Cholinergic synapse	0.000
hsa05213	Endometrial cancer	0.000
hsa04935	Growth hormone synthesis, secretion and action	0.000
hsa04926	Relaxin signaling pathway	0.000
hsa04151	PI3K-Akt signaling pathway	0.000
hsa05230	Central carbon metabolism in cancer	0.000
hsa04020	Calcium signaling pathway	0.000
hsa01522	Endocrine resistance	0.000
hsa01521	EGFR tyrosine kinase inhibitor resistance	0.000
hsa05221	Acute myeloid leukemia	0.000
hsa05235	PD-L1 expression and PD-1 checkpoint pathway in cancer	0.000
hsa04371	Apelin signaling pathway	0.000
hsa05218	Melanoma	0.001
hsa05223	Non-small cell lung cancer	0.001
hsa05163	Human cytomegalovirus infection	0.001
hsa05214	Glioma	0.001

hsa05215	Prostate cancer	0.001
hsa04510	Focal adhesion	0.001
hsa05212	Pancreatic cancer	0.001
hsa04923	Regulation of lipolysis in adipocytes	0.001
hsa05165	Human papillomavirus infection	0.001
hsa04015	Rap1 signaling pathway	0.001
hsa04213	Longevity regulating pathway - multiple species	0.001
hsa04012	ErbB signaling pathway	0.001
hsa04066	HIF-1 signaling pathway	0.001
hsa04929	GnRH secretion	0.001
hsa04210	Apoptosis	0.001
hsa04930	Type II diabetes mellitus	0.001
hsa04211	Longevity regulating pathway	0.001
hsa04724	Glutamatergic synapse	0.001
hsa04010	MAPK signaling pathway	0.001
hsa04722	Neurotrophin signaling pathway	0.002
hsa05205	Proteoglycans in cancer	0.002
hsa04919	Thyroid hormone signaling pathway	0.002
hsa05226	Gastric cancer	0.002
hsa01524	Platinum drug resistance	0.002
hsa04713	Circadian entrainment	0.002
hsa05231	Choline metabolism in cancer	0.002
hsa04933	AGE-RAGE signaling pathway in diabetic complications	0.002
hsa05220	Chronic myeloid leukemia	0.002
hsa05146	Amoebiasis	0.002
hsa04728	Dopaminergic synapse	0.003
hsa05161	Hepatitis B	0.003
hsa04931	Insulin resistance	0.003
hsa04140	Autophagy - animal	0.003
hsa05225	Hepatocellular carcinoma	0.003
hsa05418	Fluid shear stress and atherosclerosis	0.003
hsa05210	Colorectal cancer	0.004
hsa05224	Breast cancer	0.005
hsa04664	Fc epsilon RI signaling pathway	0.005
hsa04912	GnRH signaling pathway	0.005
hsa04970	Salivary secretion	0.005
hsa04917	Prolactin signaling pathway	0.006
hsa04024	cAMP signaling pathway	0.006
hsa04611	Platelet activation	0.006
hsa04921	Oxytocin signaling pathway	0.006
hsa05166	Human T-cell leukemia virus 1 infection	0.006
hsa04150	mTOR signaling pathway	0.006
hsa04062	Chemokine signaling pathway	0.008
hsa05167	Kaposi sarcoma-associated herpesvirus infection	0.008
hsa05142	Chagas disease	0.008

hsa04068	FoxO signaling pathway	0.008
hsa05100	Bacterial invasion of epithelial cells	0.008
hsa04014	Ras signaling pathway	0.009
hsa05135	Yersinia infection	0.010
hsa04662	B cell receptor signaling pathway	0.011
hsa04550	Signaling pathways regulating pluripotency of stem cells	0.012
hsa04370	VEGF signaling pathway	0.012
hsa05216	Thyroid cancer	0.013
hsa04072	Phospholipase D signaling pathway	0.014
hsa04727	GABAergic synapse	0.015
hsa04261	Adrenergic signaling in cardiomyocytes	0.015
hsa04071	Sphingolipid signaling pathway	0.015
hsa04810	Regulation of actin cytoskeleton	0.015
hsa05160	Hepatitis C	0.019
hsa04720	Long-term potentiation	0.019
hsa05414	Dilated cardiomyopathy	0.020
hsa04666	Fc gamma R-mediated phagocytosis	0.021
hsa05211	Renal cell carcinoma	0.021
hsa04750	Inflammatory mediator regulation of TRP channels	0.021
hsa04914	Progesterone-mediated oocyte maturation	0.023
hsa04972	Pancreatic secretion	0.025
hsa04973	Carbohydrate digestion and absorption	0.026
hsa04625	C-type lectin receptor signaling pathway	0.027
hsa04915	Estrogen signaling pathway	0.029
hsa05162	Measles	0.030
hsa05412	Arrhythmogenic right ventricular cardiomyopathy	0.030
hsa05132	Salmonella infection	0.031
hsa05017	Spinocerebellar ataxia	0.033
hsa04668	TNF signaling pathway	0.036
hsa04723	Retrograde endocannabinoid signaling	0.039
hsa04726	Serotonergic synapse	0.039
hsa04742	Taste transduction	0.043
hsa04911	Insulin secretion	0.043
hsa04512	ECM-receptor interaction	0.047
hsa04218	Cellular senescence	0.048

Pathways were ranked according to statistical significance.
